# Infective Endocarditis of a Rare Etiology of Streptococcus alactolyticus: A Case Report and Literature Review

**DOI:** 10.7759/cureus.57332

**Published:** 2024-03-31

**Authors:** George S Gherlan, Aura C Chicos, Ana M Veja, Mihaly Enyedi

**Affiliations:** 1 Infectious Diseases, Universitatea de Medicina si Farmacie ”Carol Davila”, Bucharest, ROU; 2 Infectious Diseases, Spitalul Clinic de Boli Infectioase si Tropicale “Dr. Victor Babes”, Bucharest, ROU; 3 Anatomy, Universitatea de Medicina si Farmacie “Carol Davila”, Bucharest, ROU

**Keywords:** bicuspid aortic valve, equinus, bovis, infective, endocarditis, streptococcus alactolyticus

## Abstract

*Streptococcus alactolyticus* is a non-motile Gram-positive, catalase-negative cocci, a part of group D *Streptococci*. In the literature, *S. alactolyticus* is documented as a causative agent of infective endocarditis, demonstrated by blood cultures in only four other cases, representing an extremely rare circumstance. Here, we describe a case of infective endocarditis due to *S. alactolyticus* in a young patient known with a bicuspid aortic valve and associated with a sigmoid precancerous polyp. The patient was also known to have blood hypertension and type II diabetes. Symptoms at the debut appeared insidiously and were non-specific: fatigue, loss of appetite, weight loss, night sweats, and fever. They lasted for the entire period of the illness with transient improvement during the courses of antibiotics. He followed more antibiotic courses prescribed for various clinical diagnoses. Each round of antibiotic treatment transitorily alleviated the symptoms, which reappeared each time after the cessation. The correct diagnosis was made only about three months after the appearance of the first clinical manifestations. This was based on ultrasound criteria (presence of vegetation and lesions of aortic cusps) and microbiological criteria (isolation of *S. alactolyticus* in blood cultures). A course of six weeks of ceftriaxone was considered the opportune antibiotic therapy. Similar to all other cases described in the literature, our patient presented important damage to the valvular tissue and required cardiac surgery to re-establish the normal function of the valve. The surgery consisted of the excision of the severely affected natural aortic valve and her replacement with a mechanical prosthetic valve. Following medical and surgical treatment, the patient is completely healed and has a normal life. Our case is noteworthy because of the scarcity of the involvement of *S. alactolyticus* in the pathogeny of infective endocarditis. This is the fifth published case with this etiology, and an overview of all five cases is provided in the article.

## Introduction

*Streptococcus alactolyticus* is a part of the *Streptococcus bovis*/*Streptococcus equinus* complex (SBSEC) that belongs to group D *Streptococci*. This complex colonizes the digestive tract in humans and animals, such as cows, goats, dogs, and poultry [1]. SBSEC members are considered commensal bacteria, but they can become the cause of various infections, especially in patients with digestive tract diseases (inflammatory bowel disease, malignancies of the intestinal wall, and colonic polyps) [1,2]. The complex is present in the intestinal flora of 5-60% of the humans tested [3]. Members of the complex are *Streptococcus equinus*, *Streptococcus infantarius* subsp. *infantarius*, *Streptococcus lutetiensis*, and *Streptococcus alactolyticus* and three subspecies of the clade *Streptococcus gallolyticus*, namely, *gallolyticus*, *macedonicus*, and *pasteurianus* [2]. The name of *Streptococcus alactolyticus* indicates that this particular member does not produce the lysis of lactose. The first description of *S. alactolyticus* was made in 1984 by a group of researchers that performed DNA hybridization and biochemical studies on a large number of *S. bovis* and *S. equinus* strains from widely varying sources in an attempt to clarify their taxonomy [4].

Infective endocarditis is an infection (bacterial, viral, or fungal) of the heart's endocardial surface, usually affecting the valves, lesser the wall's endocardium, or a septal anomaly. This is usually a severe disease with a very high potential of producing various types of complications, such as embolisation of the vegetation, valvular insufficiency of different grades, heart failure, and numerous immunological phenomena. 

*S. alactolyticus* is rarely involved in the etiology of infective endocarditis in humans. Other infections with these bacteria in humans are also rare [5]. Identifying the causative agent in infective endocarditis is crucial to manage each case correctly.

## Case presentation

We present a case of a 48-year-old male with a history of hypertension since the age of 30, known with bicuspid aortic valve, type II diabetes controlled by diet, and dyslipidemia. The patient smokes 20 cigarettes per day and consumes alcohol occasionally. He works as an engineer. The patient describes insidious symptomatology starting three months before presentation, at the beginning of September, with fatigue, loss of appetite, weight loss of about 12 kg in a period of 30 days, night sweats, fever, and lumbar pain with negative Giordano maneuver. During this frame of time, he was prescribed multiple rounds of treatment. The dermatology specialist prescribed the first two rounds with amoxicillin/clavulanic acid for seven days and then doxycycline for five days following the excision of an intradermal infected nevus from his left cheek. Then, he was prescribed oxacillin for seven days for pharyngitis, during which the fever disappeared and reappeared a few days later after stopping the antibiotic treatment. Blood tests at this time indicated mild anemia (12.3 g/dl) and inflammatory syndrome (C-reactive protein (CRP) 73.69 mg/dl, erythrocyte sedimentation rate (ESR) 50 mm/h, and fibrinogen 638 mg/dl), and he was recommended clindamycin for seven days together with methylprednisolone prescribed by a rheumatologist who suspicioned an osteoarticular disease.

At the beginning of October 2023, a thoracic and abdominal-pelvic computed tomography scan with contrast substance showed a 15 mm left paratracheal lymphadenopathy but no other significant modifications, no signs of pericarditis, no pleural effusions, and no signs of emboli.

After each round of antibiotic treatment, the fever reappeared, so he continued the medical investigations. A complete blood count was repeated and revealed a slight decrease in hemoglobin levels. He was indicated to perform a fecal occult blood test, which came out positive, and was scheduled for a colonoscopy on the 18thof October. During the procedure, three polyps were found, which were excised and sent to the pathology lab. One polyp from the sigmoid was characterized as a tubular adenoma with low-grade dysplasia, a precancerous type.

The symptomatology persisted, and on the 16th of November, blood tests showed that the inflammatory syndrome was increasing, with an ESR of 80 mm/h and a CRP of 106 mg/l. Low-grade anemia with a hemoglobin of 10.7 g/dl was present. The presumptive diagnosis of spondylodiscitis was emitted, and on the 21st of November, the patient was submitted to a magnetic resonance imaging (MRI) of the cervico-toraco-lumbar spine and hip joints. MRI showed no sign of inflammation but found L3-L4 discal circumferential protrusion and L4-L5, L5-S1 herniated disks.

He was admitted to an internal medicine ward on the 23rd of November. The laboratory tests showed mild leukocytosis with 80% neutrophils, a moderate persistent inflammatory syndrome (ESR 78 mm/h, CRP 105.90 mg/L, fibrinogen of 742 mg/dl, and ferritin 691.15 ng/ml) and positive rheumatoid factor of 15.5 IU/ml (upper limit of normal = 14 IU/ml). Three sets of blood cultures one hour apart were collected on the day of admission, and he had a transthoracic echocardiogram that raised the suspicion of endocarditis on the native aortic valve. Empiric treatment with intravenous ceftriaxone (2 g per day) was started. All three sets of blood cultures were positive for *S. alactolyticus*, susceptible to ampicillin, ceftriaxone, erythromycin, clindamycin, and vancomycin but resistant to tetracycline.

At physical examination, the only abnormal findings were the pallor of the skin and an aortic heart murmur of grade 1. Osler nodes and Janeway lesions were absent, there were no modifications to the neurological exam, and there were no signs of cardiac failure. Paraclinical tests showed no acute renal injury (normal urine dipstick). He also had an ophthalmology consult that found presbyopia, but no Roth spots on the fundoscopic exam. The patient requested to be discharged, against medical advice, on the 26th and continued antibiotherapy with ceftriaxone in a private nursing service.

**Figure 1:**
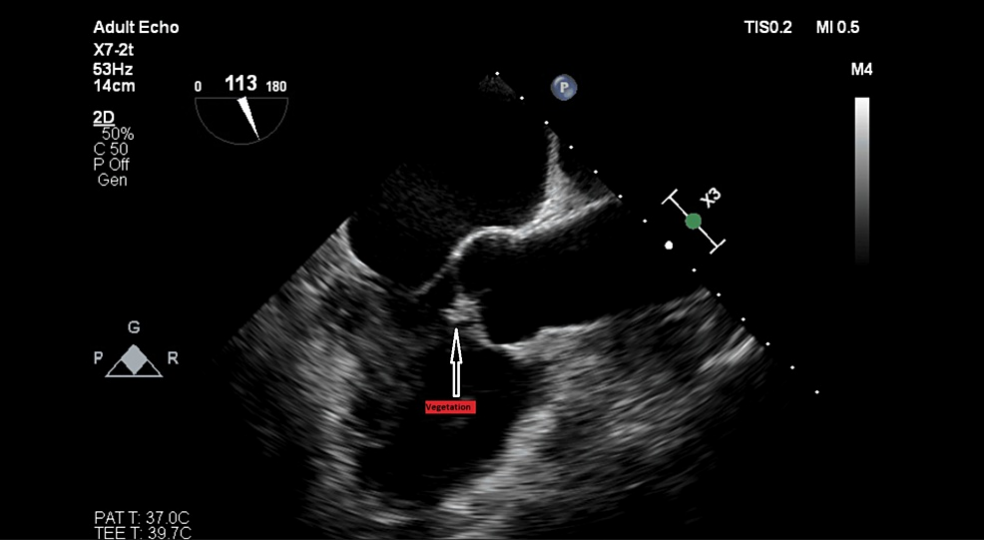
Vegetation on the bicuspid aortic valve

**Figure 2:**
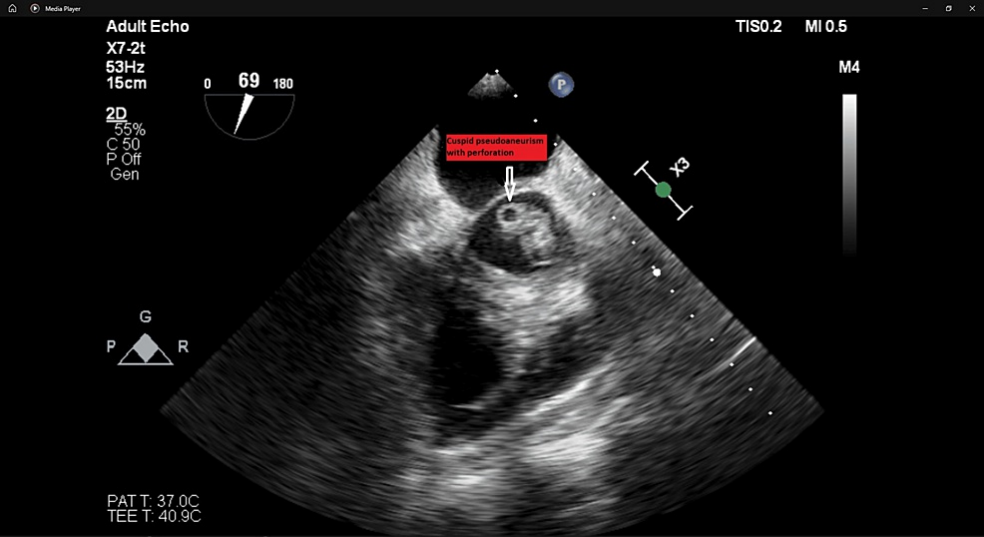
Perforation of one of the aortic cusps

On the 27th of November, he was scheduled for a transesophageal echocardiogram that described aortic bicuspid valve of type 1, with cusps at one and seven hours and calcification of junction at four hours. Both cusps had mobile, hyperechogenic masses on the ventricular side, the biggest described as having 13 mm corresponding to the fused cusp (Figure 1). Moreover, the left cusp was prolapsed. Both cusps appeared perforated, with severe regurgitation jets (Figure 2). No aortic ring abscess or pseudoaneurysm was found. Another finding was the slight dilatation of the left ventricle with hyperkinetic walls. No other lesions were identified.

At this point, we counted two major Duke criteria (positive echocardiography and cultures) and three minor criteria (bicuspid aortic valve, fever, and positive rheumatoid factor), thus confirming the diagnosis of infective endocarditis of the aortic valve.

On the 15th of December, the patient presented to our clinic for the continuation of the treatment. Two more blood samples for blood cultures were drawn. Both cultures were negative. Treatment with ceftriaxone was continued for a total of six weeks, and afterward, the patient was transferred to a surgical ward for cardiac surgery.

**Figure 3:**
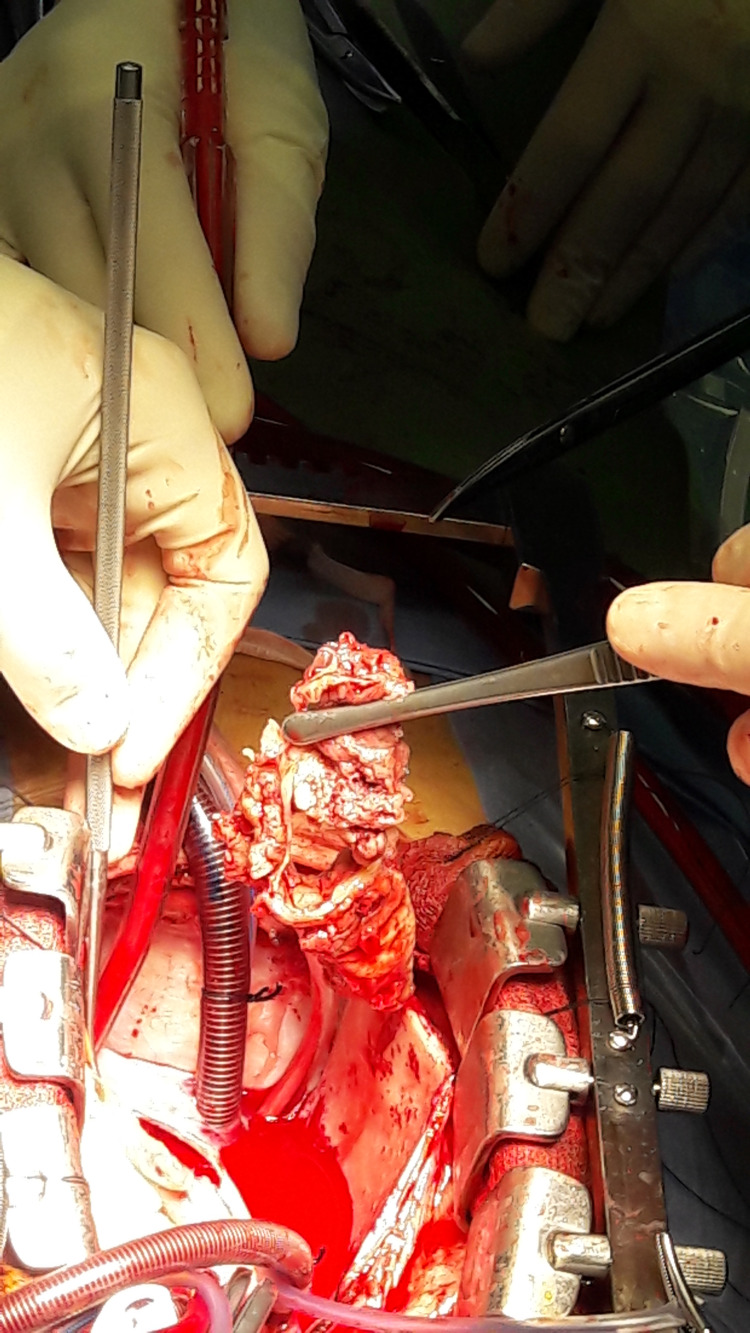
Excision of the infected aortic valve

**Figure 4:**
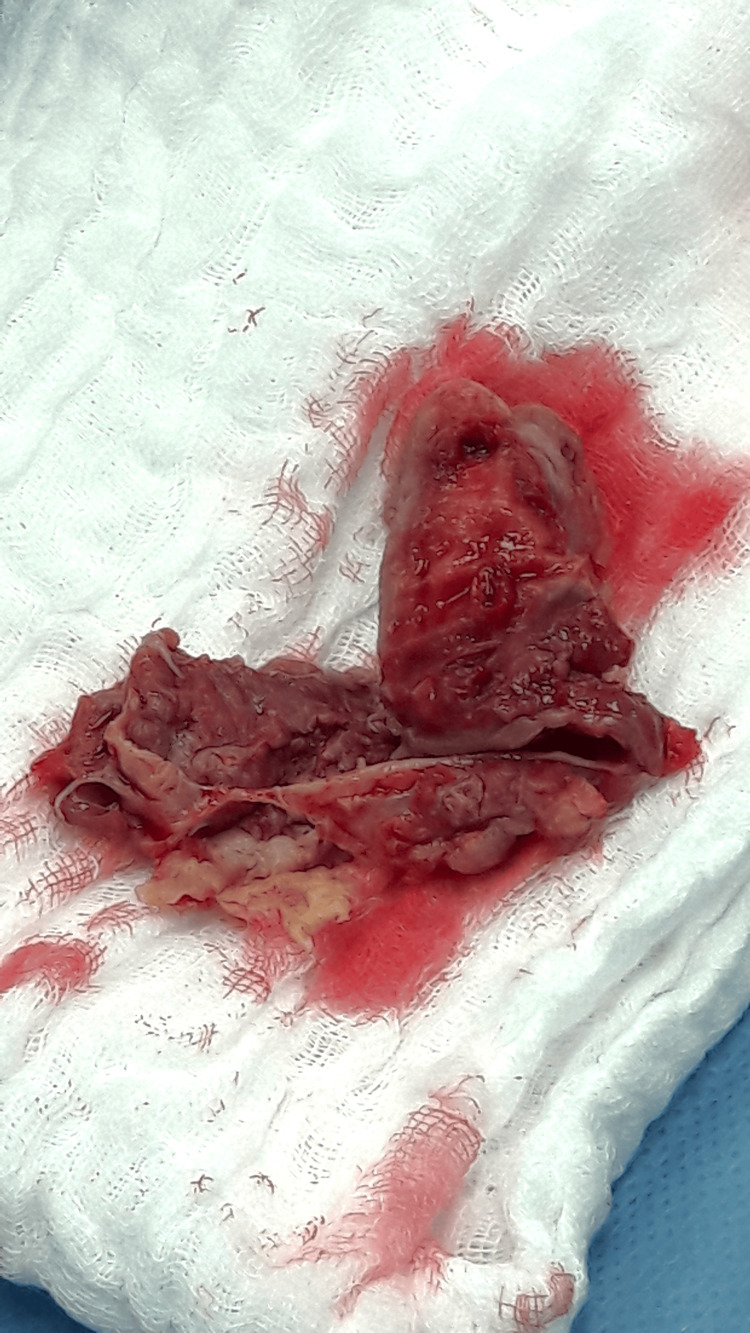
Excised aortic valve with infected thrombus

On the 15th, a follow-up set of blood cultures was ordered, and they were negative. On the 17thof January, he was transferred to another hospital for cardiac surgery. On the 18th of January, the patient underwent surgical replacement of the aortic valve with a mechanical valve (Figures 3, 4). The surgical intervention was performed under extracorporeal circulation and light hypothermia (34 degrees Celsius). A transversal aortic dissection was performed, and the aortic valve was inspected: a bicuspid valve, severely insufficient with multiple vegetations and perforated cusps. The damaged natural valve was excised and replaced with a mechanical valvular prosthesis. Closing the operation was without any hemodynamic instability, and atrial and ventricular pace wires were placed.

The surgical intervention was successful, and the patient was discharged after 10 more days of antibiotherapy. His evolution after this episode was favorable, with no complications and complete recovery in the following three months.

## Discussion

One of the most common congenital cardiac malformations is the bicuspid aortic valve, affecting 0.9-2% of the population. Complications, such as stenosis or regurgitation, may develop progressively throughout life, at a much younger age than in patients with tricuspid aortic valves. Most cases remain undiagnosed until the occurrence of infective endocarditis, dissection of the aorta (another common complication), or calcification. Infective endocarditis incidence in bicuspid aortic valve patients ranges from 10% to 30%. Moreover, in infective endocarditis, complications, such as abscesses and pseudoaneurysms or fistulae, are more commonly associated with the bicuspid aortic valve. Thus, the malformation is a significant independent predictor of complications [6,7]. All cases of infective endocarditis develop following a few common steps: bacteremia, adherence of the germ to the tissue, and eventual penetration of the germ in the endocardial tissue. The common fact in infective endocarditis is the appearance of sterile vegetation, a fibrin-platelet thrombus, or an endocardial lesion favoring the circulating germs' adherence. A pre-existent malformation and the following turbulent circulation frequently cause the appearance of this precursor of endocarditis.

*S. alactolyticus* is a part of the SBSEC, a non-enterococcal group D *Streptococci* colonizing animals and the human intestinal tract. This *Streptococci *genus is part of lactic acid bacteria [2]. *S. alactolyticus* predominates among the culturable lactic acid bacteria and was also described as the main bacteria in mozzarella soft cheese (in a study on cheese samples from Baghdad) [8] and can also be found in raw milk (also in Baghdad) [9].

SBSEC is associated with bacteremia, infective endocarditis, and colorectal cancer, but members of this group are also described as predominant species in food fermentations, being beneficial for the quality of fermented products. Being in the same family, *S. alactolyticus* shares pathophysiological similarities with *S. bovis*, although *S. alactolyticus* is rarely described as pathogenic in humans. Throughout the years, it has been well established that SBSEC is strongly linked to colonic cancer or intestinal diseases associated with inflammation or morphology changes. SBSEC colonization in patients with colorectal cancer is higher than in persons without colorectal cancer [10].

Most of the time, the risk factors associated with endocarditis are investigated post-diagnosis. In cases with positive blood cultures for bacteria from the SBSEC group, the patients are investigated through a colonoscopy or checked for dental cavities. Our patient was diagnosed with colonic polyps before being given the diagnosis of endocarditis, and it was found that he had a precancerous type of polyp. It should be noted that his father was diagnosed at 56 years old with advanced-stage colonic cancer with liver metastases. According to the screening protocol for colonic cancer, the patient should have started the screening at 46 years.

Table 1 shows published cases of infective endocarditis caused by *S. alactolyticus*.

**Table 1:** Published cases of infective endocarditis caused by Streptococcus alactolyticus highlighting age, country of provenience, underlying condition, affected valves, and preferred treatment

Year	Age, sex	Country	Underlying conditions	Affected valves	Positive hemoculture	Antibiogram/treatment	Reference
2021	69, M	Italy	Liver steatosis, colon cancer (30 years ago)	Mitral valve	S. alactolyticus	Treatment with ceftriaxone and gentamicin	[5]
2020	64, M	Greece	Hypertension, type II diabetes, mitral valve prolapse	Mitral valve	S. alactolyticus	Resistance to gentamicin. Treatment with ceftriaxone	[11]
2019	64, M	Turkey	Coronary artery bypass graft surgery	Aortic and mitral valves	S. alactolyticus	Treatment with ceftriaxone	[12]
2016	65, F	USA	Hypertension, hyperlipidemia, hypertrophic cardiomyopathy, mitral regurgitation. Recent dental procedure (cleaning)	Aortic and mitral valves	S. alactolyticus	Treatment with ceftriaxone	[13]

In the case of infective endocarditis due to *S. alactolyticus* described in 2019, this was susceptible to tetracycline [12]. The antibiotic susceptibility testing of *S. alactolyticus* from our patient’s blood cultures showed resistance to tetracycline. Studies on animal feces have reported changes in antimicrobial resistance. A Korean study on ruminant animals specifically studied SBSEC bacteria and their antibiotic sensitivities, showing intermediary susceptibility to tetracycline, erythromycin, and clindamycin [14]. Variable resistance rates have been reported for clindamycin, erythromycin, tetracycline, and levofloxacin [3]. The first case of vancomycin-resistant *S. alactolyticus* in raw milk was described in 2016 [9].

## Conclusions

The diagnosis of infective endocarditis is sometimes delayed because of its insidious and non-specific clinical manifestations. Therefore, it is very important to be aware of this possibility. In our patient's case, another factor that contributed to the delay was the inappropriate use of antibiotics.

Although considered a commensal bacteria normally colonizing the digestive tract of humans, *S. alactolyticus* can produce, in rare situations, bacteriemia and infections in various sites. Such instances should lead to investigations to identify the entry gates of these germs.

*S. alactolyticus* is a rare cause of infective endocarditis in humans, but all the cases described in the literature were severe and required surgery to repair the damage caused by the infection. All described patients had underlying heart medical conditions except one. Ceftriaxone was the preferred antibiotic in all cases.

Further extensive epidemiological studies on this subject would be beneficial for a better understanding of the real prevalence of this etiology in the landscape of infective endocarditis.
